# Clinical audit as a catalyst for quality improvement in healthcare systems: strategic insights from a systematic review and SWOT–TOWS analysis

**DOI:** 10.1136/bmjoq-2026-004162

**Published:** 2026-10-21

**Authors:** Ansif Pallath Majeed, Niyaz Panakaje, Shajitha Thekke Veettil, Kiran Harikumar, Saurav Sathish Kumta, Hanan Al Mujalli, Sharifullah Khan, Abdul Ali Shah, Noora Jassim Alkubaisi

**Affiliations:** 1 Department of Management, Yenepoya Institute of Arts, Science, Commerce and Management, Yenepoya (Deemed to be University), Mangaluru, Karnataka, India; 2 Department of Clinical Affairs, Primary Health Care Corporation, Doha, Qatar; 3 YEN-REFINED, Yenepoya Institute of Arts, Science, Commerce and Management, Yenepoya (Deemed to be University), Mangaluru, Karnataka, India

**Keywords:** Healthcare quality improvement, Patient-centred care, Audit and feedback

## Abstract

**Background:**

Clinical audit is fundamental to clinical governance and improving healthcare quality and safety. However, its potential for sustained quality improvement is often underutilised. This study explores how clinical audits act as catalysts for quality improvement in primary care using a strategic analytical approach.

**Methods:**

The study involved a qualitative systematic review of peer-reviewed literature from January 2015 to March 2024. Structured database searches and predefined inclusion criteria were used to identify relevant studies, and thematic analysis was employed to synthesise data. The findings were mapped onto a SWOT (Strengths, Weaknesses, Opportunities, Threats) framework, followed by development of a TOWS matrix to convert insights into actionable strategies.

**Results:**

35 studies met the inclusion criteria, covering diverse primary care settings and study designs. Evidence consistently showed that clinical audits improved adherence to guidelines, interdisciplinary collaboration and patient care processes. Key strengths included better clinical practice, knowledge sharing and teamwork. Opportunities were found in integrating audit results into clinical systems, supporting policy development and optimising resources. Weaknesses identified were reduced clinical ownership, fear of blame or litigation, and health information system limitations. Threats included professional scepticism, institutional inertia and time constraints. The Threats, Opportunities, Weaknesses, and Strengths (TOWS) analysis underscored the importance of non-punitive audit environments, leadership engagement and transparent communication to enhance audit impact.

**Conclusions:**

Clinical audit remains a vital tool for quality improvement in primary care. When supported by strong organisational structures, audits can evolve from compliance tasks to dynamic instruments for organisational learning and patient-centred care. The SWOT–TOWS analysis offers strategic guidance for strengthening audit processes and achieving lasting improvements in clinical practice.

**WHAT IS ALREADY KNOWN ON THIS TOPIC**

- Clinical audits are widely used to improve healthcare quality and patient safety by measuring clinical practice against standards. However, audits often face well-documented challenges such as low clinician engagement, resource constraints, and a culture of blame or fear of litigation that can limit their impact on sustained quality improvement.

**WHAT THIS STUDY ADDS**

- By synthesising evidence from 35 studies, this systematic review is the first to apply a combined Strengths, Weaknesses, Opportunities, Threats–TOWS framework to clinical audits. It identifies critical internal weaknesses (eg, poor data systems, lack of ownership) and external threats (eg, institutional inertia) and provides actionable strategies to address them, for example, implementing non-punitive feedback mechanisms, strengthening leadership support for audits and integrating audit findings into policy and training. These findings offer a novel, evidence-based strategic roadmap to enhance the effectiveness of clinical audit programmes.

**HOW THIS STUDY MIGHT AFFECT RESEARCH, PRACTICE OR POLICY**

- The strategic recommendations presented can help healthcare organisations and policymakers strengthen their clinical governance and quality improvement efforts. Adopting the recommended interventions (such as fostering an open ‘just culture,’ dedicating resources for audit follow-up and aligning audit outcomes with organisational learning) may improve frontline staff engagement in audits, promote continuous improvement in patient care and inform future research on implementing quality and safety frameworks in practice.

## Introduction

Clinical audits have become essential for improving patient care by systematically evaluating clinical practices against established standards. Over the past decade, increased emphasis on quality, safety and efficiency in healthcare has further amplified the importance of audit and feedback mechanisms.^1 2^ Clinical audits are widely recognised for promoting evidence-based practice, enhancing patient safety and supporting continuous quality improvement across diverse healthcare settings.^3 4^

Recent studies demonstrate that structured audit programmes improve compliance with clinical guidelines, reduce complications and optimise resource utilisation.^5 6^ Audits also foster interdisciplinary collaboration, facilitate knowledge sharing and support professional development among healthcare teams.^7 8^ However, the effectiveness of clinical audits is frequently constrained by persistent challenges, including data quality limitations, resource constraints and organisational resistance to change.^9 10^ Additional barriers such as outdated information systems, hierarchical organisational structures and fear of litigation have also been shown to hinder meaningful engagement and sustained improvement.^11 12^

To better understand these complex and interacting influences, this study applies a Strengths, Weaknesses, Opportunities, Threats (SWOT)–TOWS analytical framework. SWOT analysis systematically examines internal strengths and weaknesses and external opportunities and threats, providing a structured way to map contextual factors that influence performance and implementation.^13^ The TOWS matrix builds on this framework by explicitly aligning these factors to generate strategic response options, thereby translating descriptive findings into actionable directions for improvement.^14^ This combined approach is particularly suitable for synthesising heterogeneous evidence and for supporting strategic decision-making in healthcare quality improvement contexts.

Accordingly, this study aims to identify the key strengths, weaknesses, opportunities and threats associated with clinical audit programmes and to use the TOWS matrix to derive evidence-informed strategic recommendations to optimise the impact of clinical audits in contemporary healthcare settings.

Importantly, the SWOT–TOWS framework applied in this study is not merely descriptive or theoretical; rather, it represents a strategy generating and implementation-oriented approach that directly translates contextual analysis into actionable, evidence-based interventions. By moving beyond identification of barriers and facilitators to explicitly link them with targeted strategies, this approach enhances the practical relevance, applicability and impact of clinical audit in real-world healthcare settings. To our knowledge, this represents the first systematic application of a SWOT–TOWS framework to the synthesis of clinical audit literature.

## Methodology

We conducted a systematic review in accordance with Preferred Reporting Items for Systematic Reviews and Meta-Analyses (PRISMA) guidance to examine how clinical audits contribute to healthcare quality improvement and to identify factors relevant to SWOT–TOWS analysis. Searches were undertaken in PubMed/MEDLINE, Web of Science, the Cochrane Library and Mendeley for peer-reviewed English-language publications from January 2015 to March 2024. Search terms were organised across five domains: clinical audit, healthcare quality improvement, SWOT analysis, TOWS matrix, and process or outcome factors. Key terms included ‘benchmarking,’ ‘patient safety,’ ‘evidence-based practice,’ ‘stakeholder engagement,’ ‘continuous monitoring,’ ‘change management’ and ‘performance indicators,’ combined using Boolean operators. Full database-specific search strings, limits and syntax adaptations are provided in online supplemental table 1.

After merging search results, 1376 duplicate records were removed using reference management software and manual verification. This left 4218 unique records for title and abstract screening. Two reviewers independently screened records, and a third reviewer resolved discrepancies. At this stage, 3892 records were excluded because they were unrelated to clinical audit, quality improvement or relevant outcomes. Full texts were retrieved for 326 articles and assessed independently against predefined eligibility criteria. Of these, 291 were excluded for reasons including lack of focus on clinical audit, non-healthcare setting, ineligible study design or unavailable full text. Overall, 35 studies met the inclusion criteria and were included in the qualitative synthesis. The study selection process is summarised in the PRISMA flow diagram (figure 1).

**Figure 1:**
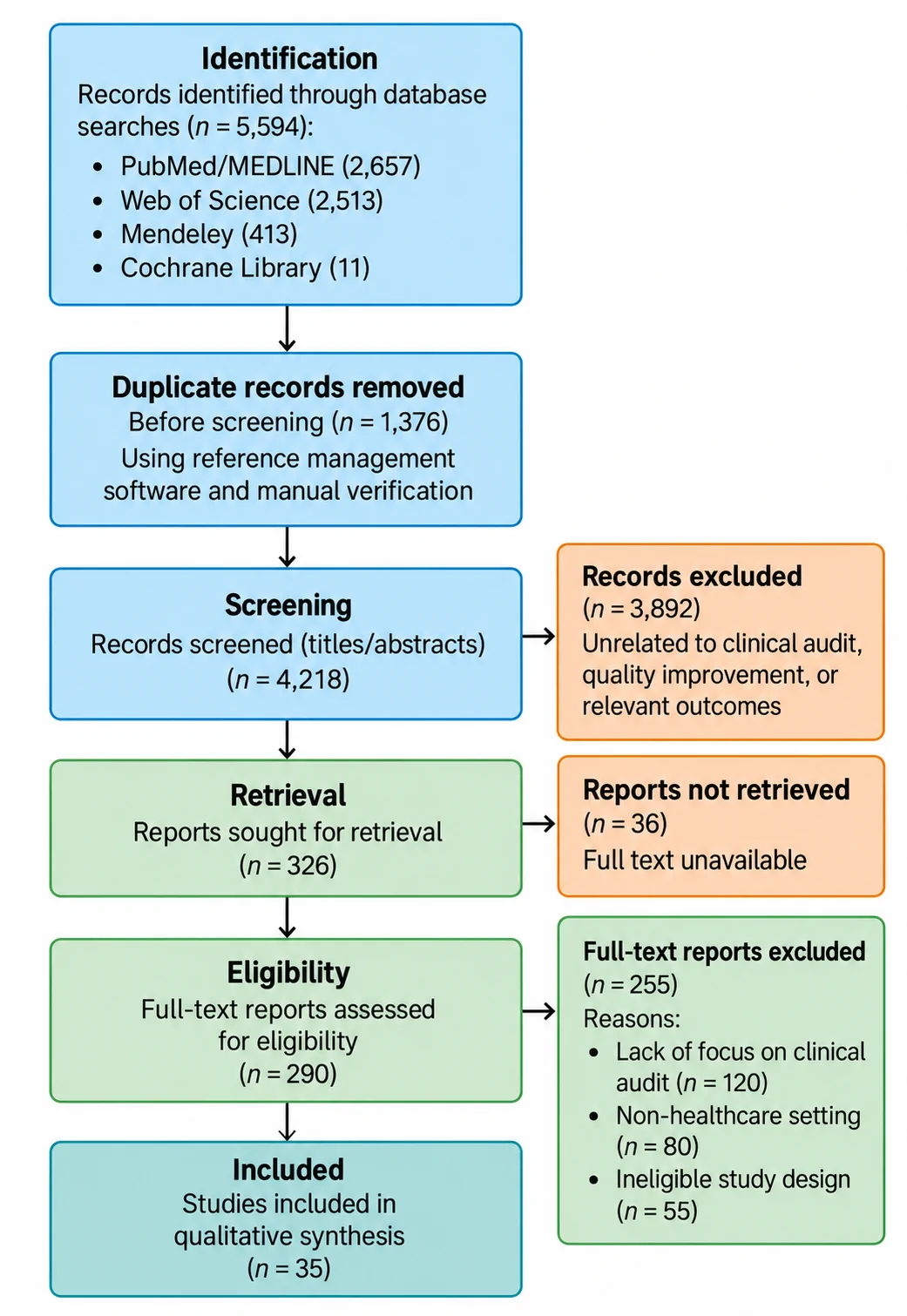
PRISMA flow diagram. PRISMA, Preferred Reporting Items for Systematic Reviews and Meta-Analyses.

Eligible studies were peer-reviewed articles published in English between 2015 and 2024 that addressed clinical audit processes, implementation or outcomes within healthcare management and provided sufficient methodological detail for appraisal. We included quantitative audit outcome studies, qualitative audit process studies, mixed-methods and case studies, healthcare management or policy analyses, and studies explicitly describing audit barriers or enablers relevant to SWOT–TOWS analysis.

Studies were excluded if they did not specifically examine clinical audit impact or processes, were conducted outside healthcare settings, lacked full-text availability or were opinion pieces, commentaries or editorials without empirical data.

### Data extraction and synthesis

For each included study, two reviewers independently extracted relevant information from the results and discussion sections, including reported benefits, challenges, outcomes and contextual factors associated with clinical audit implementation. Extracted findings were converted into a common narrative format to support synthesis across quantitative, qualitative, mixed-methods and policy-focused studies. Quantitative findings, such as changes in guideline adherence or patient outcomes, were interpreted alongside qualitative evidence on barriers, facilitators and implementation context rather than pooled statistically.

The synthesis used a framework-guided approach. Extracted findings were first grouped into preliminary themes and then mapped to SWOT domains: Strengths and Weaknesses were defined as internal factors within the audit programme or organisational context, while Opportunities and Threats were defined as external factors arising from the broader system or environment. Positive or enabling factors were classified as Strengths or Opportunities, and negative or constraining factors as Weaknesses or Threats. Disagreements in categorisation were resolved through discussion, with a senior reviewer consulted where required. Decisions were documented in an internal synthesis table to support transparency.

The final SWOT factors were then used to develop a TOWS matrix. The review team systematically paired Strengths with Opportunities to generate S–O strategies, Weaknesses with Opportunities to generate W–O strategies, Strengths with Threats to generate S–T strategies and Weaknesses with Threats to generate W–T strategies. Each proposed strategy was checked against the supporting evidence and refined through consensus to ensure that recommendations were practical, evidence-informed and aligned with the review findings. Online supplemental table 2 summarises the included study types and their contribution to the SWOT–TOWS synthesis.

### Mixed-methods synthesis, SWOT mapping and quality appraisal

We used a convergent integrated synthesis approach to combine evidence from quantitative, qualitative, mixed-methods, case-based and policy-focused studies. Relevant statistical results, qualitative themes and narrative observations were transformed into a common textual format so that outcome evidence, such as improvements in compliance or patient outcomes, could be interpreted alongside contextual evidence on barriers, facilitators and implementation conditions. This enabled numerical and qualitative evidence to contribute equally to the synthesis rather than being analysed separately by study design.

Two reviewers independently extracted and coded findings from each included study, focusing on clinical audit effects, barriers and facilitators. The extracted findings were then mapped to the predefined SWOT domains using a directed framework approach. Internal positive factors were classified as Strengths, internal negative factors as Weaknesses, external facilitating factors as Opportunities and external constraining factors as Threats. Positive or enabling findings, such as effective practices, supportive culture or policy incentives, were assigned to Strengths or Opportunities depending on their source, whereas negative or hindering findings, such as resource gaps, cultural resistance or regulatory barriers, were assigned to Weaknesses or Threats.

Mapping decisions were first made independently and then discussed by the review team to resolve differences. Where a theme could fit more than one category, the team considered the context of the finding and the interpretation of the original study authors before assigning the dominant classification. For example, ‘lack of senior management support’ was classified as a Threat because it reflected a broader leadership climate beyond the immediate control of the audit team, whereas ‘staff fear of blame’ was classified as a Weakness because it reflected internal team or institutional culture. Any remaining disagreements were resolved by discussion, with a senior reviewer consulted when required. Online supplemental table 3 provides illustrative examples of how extracted themes were mapped to SWOT categories.

The mapping process was primarily deductive because the SWOT categories were defined as a priority, but it allowed interpretive flexibility where unexpected findings required refinement of category interpretation. All extracted findings were meaningfully accommodated within the four SWOT domains, so no additional categories were introduced. Decisions were documented in an internal synthesis table to maintain transparency, consistency and reproducibility. This framework-guided approach is consistent with qualitative evidence synthesis methods, including framework or best-fit synthesis, and addresses PRISMA 2020 expectations for transparent reporting of data preparation and synthesis methods.

### Quality appraisal

The methodological quality of included studies was assessed to support interpretation of findings. Given the heterogeneity of study designs, we used an adapted appraisal approach drawing on criteria relevant to quantitative, qualitative and mixed-methods research. Key domains included clarity of study objectives, appropriateness of design, data collection methods, transparency of analysis and relevance to clinical audit practice. Quality appraisal informed the weight and credibility of evidence contributing to the SWOT and TOWS synthesis but was not used as an exclusion criterion.

### Data synthesis and strategic framework application

After data extraction, findings from the included studies were systematically organised using the SWOT framework. Relevant evidence was classified as either internal factors Strengths and Weaknesses or external factors Opportunities and Threats according to the nature and direction of each finding. This structured, framework-based approach enabled a comprehensive synthesis of heterogeneous evidence without applying formal qualitative coding. The definitions and application of each SWOT and TOWS domain are summarised in table 1.

**Table 1:** Definition of SWOT and TOWS

Component	Definition
Strengths	Internal factors that support effective clinical audit implementation, including improvements in clinical care processes, adherence to evidence-based guidelines, interdisciplinary teamwork, shared learning opportunities, effective communication and positive patient satisfaction outcomes.
Weaknesses	Internal limitations that may constrain audit effectiveness, including reduced clinical ownership, fear of blame or litigation, hierarchical and territorial barriers, limited resources, and weaknesses in clinical information systems affecting data availability and feedback.
Opportunities	External enabling factors that can enhance audit implementation, including integration with clinical information systems, improved feedback mechanisms, influence on policy and guidelines, workforce skill development, resource optimisation and the development of locally relevant standards of care.
Threat	External barriers that may hinder sustained engagement in audit activities, including scepticism toward audits, reluctance to provide peer feedback, fear of intimidation or reputational harm, institutional inertia, time constraints, and professional isolation.
S–O	Strategies that leverage internal strengths to capitalise on external opportunities, such as using strong teamwork and learning cultures to integrate audit outputs into clinical systems and organisational policies.
W–O	Strategies that use external opportunities to overcome internal weaknesses, including targeted training and system improvements to enhance clinical ownership, close skill gaps and improve data availability.
S–T strategies	Strategies that use existing strengths to mitigate external threats, such as leveraging evidence of improved care and leadership engagement to counter resistance, scepticism and institutional inertia.
W–T strategies	Strategies aimed at minimising internal weaknesses and reducing vulnerability to external threats, including fostering a non-punitive audit culture, clarifying standards and allocating protected time to address workload pressures and fear of blame.

### SWOT-based strategy formulation (TOWS matrix)

Following the thematic synthesis and SWOT categorisation of findings (described in table 2), we engaged in a structured strategy formulation step using a TOWS matrix approach. The aim of this step was to translate the identified SWOT factors into practical strategic recommendations for optimising clinical audit implementation in healthcare. Two reviewers (the same who led the SWOT mapping) first collated all Strengths (S), Weaknesses (W), Opportunities (O) and Threats (T) factors identified across the included studies. Working with this comprehensive list, we then systematically examined pairings of internal and external factors to generate potential strategies across four categories: S–O strategies (leveraging strengths to capitalise on opportunities), W–O strategies (using opportunities to address or overcome weaknesses), S–T strategies (using strengths to mitigate or avoid threats) and W–T strategies (minimising weaknesses to reduce vulnerability to threats). This process was primarily deductive or concept-driven applying the classic TOWS matrix framework to our data, but it remained closely anchored to the empirical evidence (thus incorporating an inductive element) by ensuring that each proposed strategy directly drew on specific SWOT factors identified from the studies.

**Table 2:** Included studies and their key characteristics

Characteristic	Summary of included studies (n=35)
Publication years	2015–2024
Study designs	~1/3 quantitative (observational or interventional audits), ~1/4 qualitative (interview or survey-based studies), ~1/5 mixed-methods, remainder policy/management analyses
Study populations	Primarily healthcare providers and teams engaged in clinical audits (physicians, nurses, managers); some studies also examined patient outcomes or records
Interventions/exposures	Clinical audit processes or audit-and-feedback interventions (local audit cycles, national audit programmes, etc)
Settings	Predominantly primary care settings; some hospital or system-level contexts
Geographic context	International (studies from multiple countries across Europe, North America, Asia, Middle East)

### Practical process

The two reviewers independently drafted initial strategy ideas for each TOWS category, referencing the relevant SWOT factors (and their source studies) that informed each idea. For example, if our SWOT analysis identified a Strength such as ‘presence of dedicated audit-trained staff’ and an Opportunity like ‘availability of external funding for quality improvement,’ one S–O strategy we formulated was ‘Leverage dedicated audit-trained staff to secure and effectively utilize external QI funding for sustained audit cycles.’ This candidate strategy explicitly links an internal advantage to an external enabler. Similarly, using an identified Weakness (eg, ‘insufficient protected time for audits’) and an Opportunity (eg, ‘national policy support for audit initiatives’), we derived a W–O strategy like ‘Advocate for policy-driven funding to alleviate time constraints, integrating audit duties into regular workflows with policy backing.’ We repeated this approach for S–T and W–T pairings as well, such that each strategy in our results is traceable to specific SWOT factors from the data.

### Consensus and validation

After independently generating these strategic options, the two reviewers and a third senior team member held a series of consensus meetings to review and refine the TOWS strategies. During these discussions, we verified that each strategy was logical, systematically derived and justifiable based on the evidence. We also removed or merged any overlapping strategies and ensured clarity of wording. We kept a documented mapping of which SWOT factors (and corresponding study references) informed each strategy to maintain an audit trail. This collaborative formulation process resembles an ‘abductive’ integration: we used a theoretical framework (TOWS) guided by data-derived insights to create something new actionable strategies that were not explicitly stated in the primary studies but are well-grounded in their findings.

### Design and synthesis approach

This study was conducted as a systematic review of heterogeneous evidence, including qualitative studies, quantitative audit outcome studies, mixed-methods research and case studies. Although the analytic techniques applied were qualitative in nature, the review is not positioned as a qualitative systematic review per se, as the evidence base extended beyond qualitative study designs.

Given the diversity of included methodologies and the strategic, implementation-focused aim of the review, we adopted a convergent integrated synthesis approach. Findings from all included studies were first extracted and, where necessary, transformed into narrative form (eg, reported benefits, barriers, contextual enablers and risks associated with clinical audit implementation). Quantitative results (such as pre–post audit outcomes) were not pooled statistically but were interpreted qualitatively to inform higher-order conceptual themes.

These synthesised findings were subsequently organised using a SWOT framework, which served as a theory-informed analytical structure for integrating diverse evidence types. This framework is well suited to mixed-evidence reviews where the objective is to understand systemic enablers and constraints rather than to estimate effect sizes. The SWOT findings were then translated into strategic recommendations using a TOWS matrix, representing an evidence-informed interpretive step rather than a statistical inference.

This approach allows heterogeneous evidence to be synthesised coherently while maintaining transparency about the analytical logic and limitations of cross-design integration.

### Framework integration

To connect our findings with established implementation science, we selected the SWOT–TOWS framework as a guiding heuristic for our synthesis. The decision was informed by the desire to integrate diverse evidence within a structured strategic planning lens. We acknowledge that our synthesis is best described as a mixed-methods (convergent) systematic review; while the included studies were of various designs (qualitative, quantitative, mixed), our analysis was qualitative, using thematic synthesis followed by directed categorisation into SWOT domains and interpretative strategy generation via TOWS matrix. This integrative approach allowed us to draw on implementation science principles*: for example, differentiating ‘internal’ Strengths/Weaknesses versus ‘external’ Opportunities/Threats parallels the emphasis on inner versus outer context in frameworks like the Consolidated Framework for Implementation Research (CFIR). We then used the TOWS matrix quadrant logic to craft strategies that leverage or address these factors in combination, linking back to documented implementation strategies (eg, those outlined in the ERIC (Expert Recommendations for Implementing Change) compilation or recommended by Grol & Grimshaw) where applicable. Thus, our strategic recommendations emerged from a hybrid process: they were grounded in the empirical evidence (SWOT factors drawn from included studies), but the act of formulating them involved an interpretative application of established implementation concepts (like champions, incentives, feedback, training) which are well-documented in the implementation science literature.

## Results

The review included 35 studies published between 2015 and 2024. In terms of study design, 12 were quantitative studies (eg, audits reporting statistical outcomes or before–after comparisons), 8 were qualitative studies (eg, interview-based or survey-based studies exploring audit processes) and 6 were mixed-methods studies combining quantitative outcomes with qualitative contextual data. The remaining 9 studies comprised policy or management analyses of clinical audit programmes at a systems level. This diversity of designs ensured that both empirical outcomes and contextual insights could inform the SWOT analysis. The included studies also varied in their populations (primarily healthcare professionals involved in clinical audits, with some examining patient outcomes), interventions (focused on clinical audit processes or structured audit-and-feedback programmes as quality improvement strategies), settings (predominantly primary care clinics, with some in hospital departments or multi-site/national programmes) and geographic locations (studies from Europe, North America, Asia and the Middle East), providing a broad context for our synthesis.

### Geographic and economic context

The included studies spanned multiple world regions, but the majority were conducted in high-income health system settings. In particular, 26 of the 35 included studies (74%) took place in high-income countries (HICs), such as the UK, Northern/Western Europe, North America or high-income Middle Eastern countries, which typically have well‐established audit infrastructures and abundant resources. A smaller subset of 7 studies (20%) were conducted in upper middle-income countries (eg, upper-middle-income countries settings in East Asia and Central Europe), and only 2 studies (6%) were from lower middle-income countries (LMICs). None of the included studies were set in low-income countries. We have provided a detailed breakdown of each study’s country and corresponding World Bank income classification in online supplemental table 2 for complete transparency. This skew toward economically advanced settings is an important contextual factor when interpreting our findings.

#### SWOT analysis findings

Using a structured, framework-based synthesis, we examined the extracted findings from all included studies and organised them into a SWOT framework. Information describing internal facilitators or limitations of clinical audit processes was classified as Strengths or Weaknesses, while factors arising from the broader organisational or policy environment were classified as Opportunities or Threats. This classification was refined through iterative team discussion to ensure consistency, with consensus reached on the final assignment of each factor to a SWOT domain. The resulting SWOT framework provides an overview of the key factors influencing the effectiveness of clinical audit in healthcare (summarised in figure 2).

**Figure 2:**
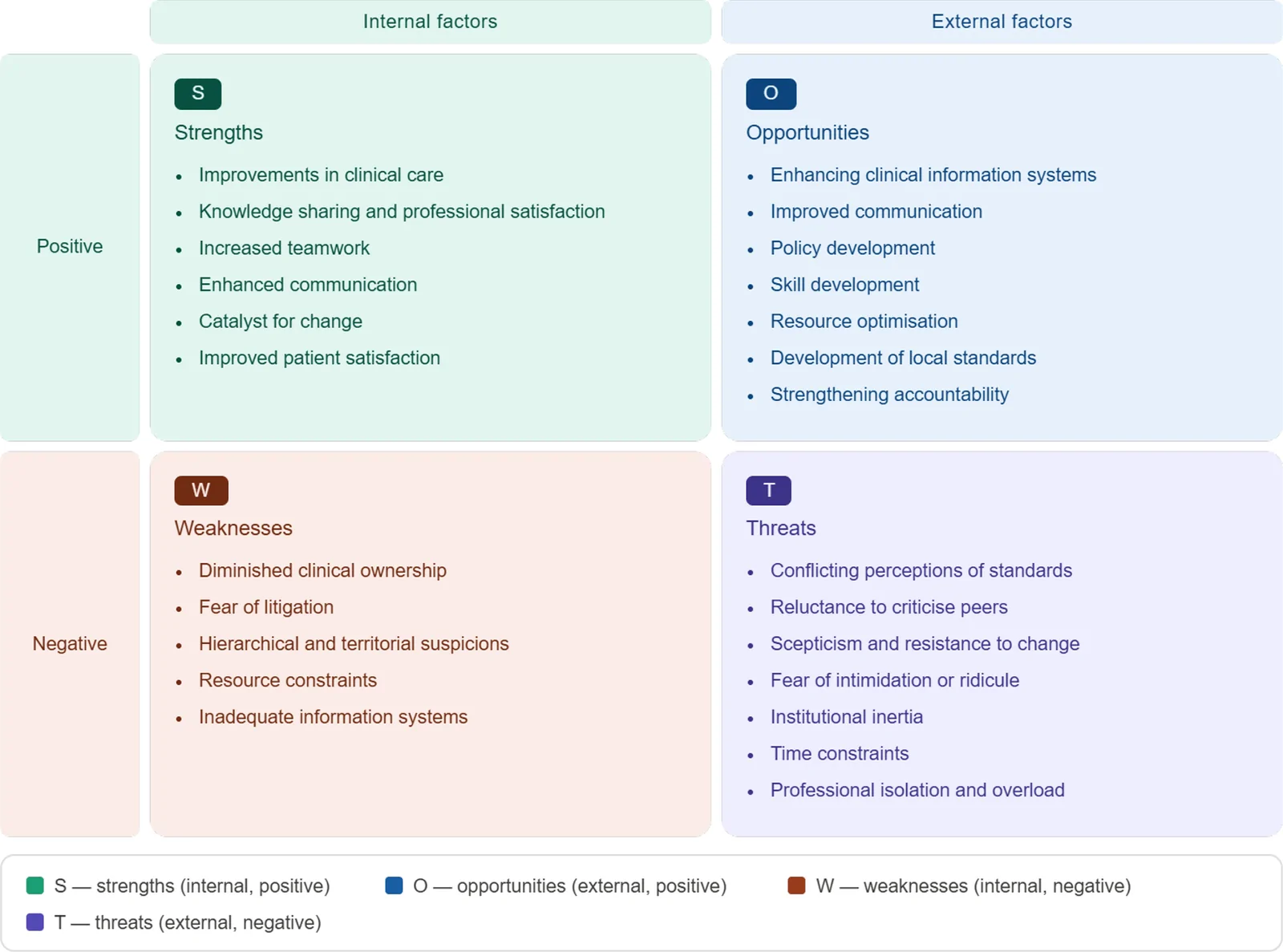
SWOT analysis of clinical audit in healthcare. Key Strengths (improvements in care, shared learning, teamwork, communication, change momentum, patient satisfaction) and Weaknesses (low ownership, fear of litigation, hierarchical barriers, resource gaps, poor info systems) are internal factors identified. External Opportunities (enhancing information systems, better feedback communication, policy influence, skill building, resource optimisation, local standards, accountability) and Threats (conflicting standards perceptions, reluctance to critique, scepticism, intimidation, inertia, time limits, professional isolation) were also recognised. SWOT, Strengths, Weaknesses, Opportunities, Threats.

#### TOWS-derived strategies

Building on the SWOT analysis, we developed a TOWS matrix to align internal and external factors and derive practical strategies (presented in figure 3). This matrix pairs Strengths with Opportunities (S–O strategies) to amplify positive influences, leverages Opportunities to address Weaknesses (W–O strategies) and Strengths to mitigate Threats (S–T strategies) and minimises Weaknesses to reduce Threats (W–T strategies). Each strategic recommendation is directly grounded in the evidence from the SWOT synthesis. These strategies are described in detail in the next section of the Results, offering a set of actionable insights that healthcare organisations can apply to bolster the role of clinical audits in driving quality improvement.

**Figure 3:**
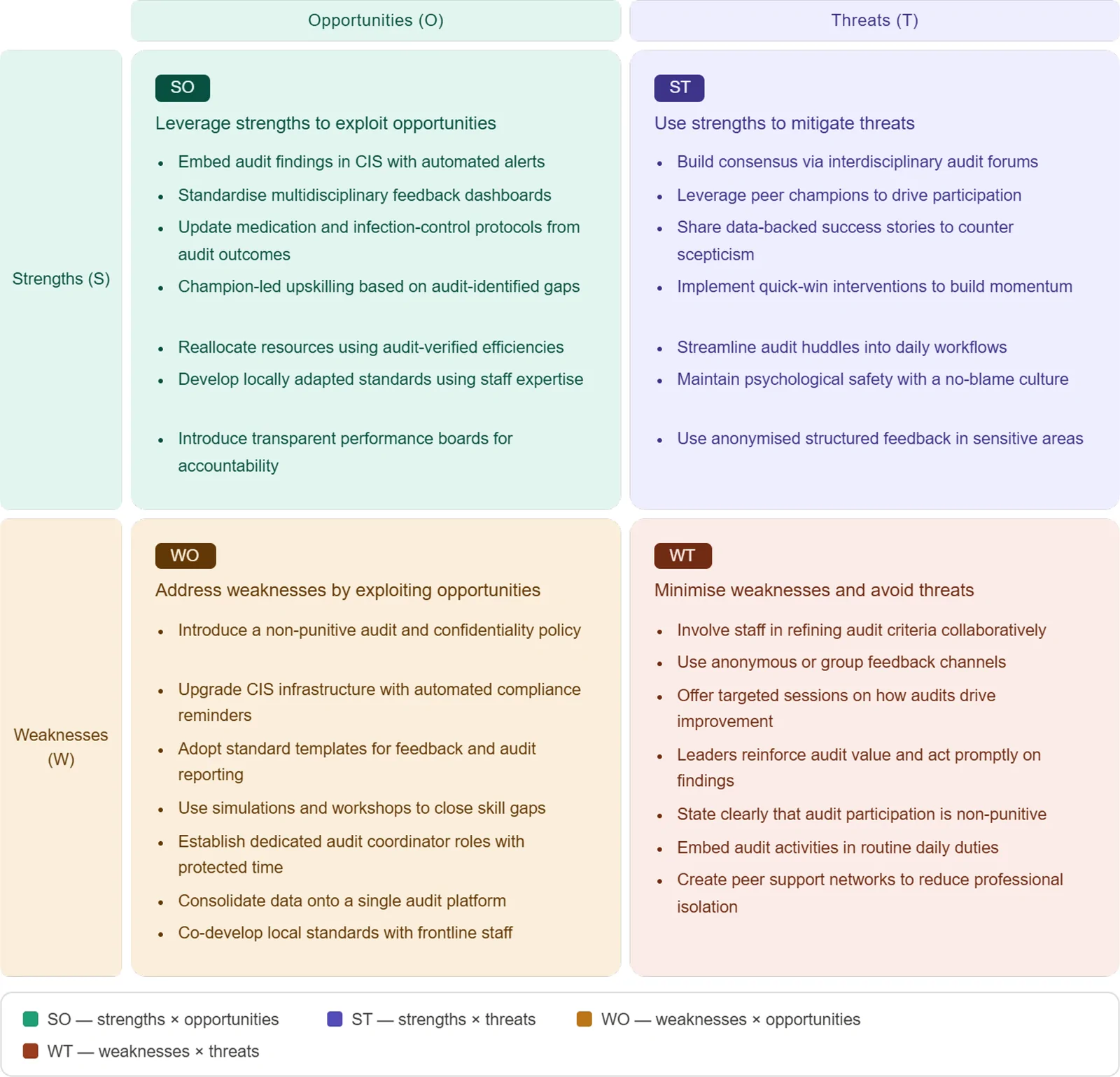
TOWS matrix for strategic planning*.* This matrix aligns the internal factors (Strengths, Weaknesses) with external factors (Opportunities, Threats) to generate strategies. S–O strategies leverage strengths to capitalise on opportunities (eg, using effective audit processes to improve information systems). W–O strategies use opportunities to address weaknesses (eg, providing training to overcome skill gaps). S–T strategies use strengths to mitigate threats (eg, using strong leadership and evidence of success to counteract scepticism and inertia). W–T strategies minimise weaknesses to avoid or reduce threats (eg, improving clarity and support to prevent miscommunication and intimidation)*.* Simplified TOWS matrix outlining key strategies to strengthen clinical audit implementation. The strategies are organised into four domains: SO strategies, which use existing strengths to capitalise on opportunities; ST strategies, which apply strengths to mitigate potential threats; WO strategies, which address weaknesses by leveraging available opportunities; and WT strategies, which aim to minimise weaknesses and reduce exposure to threats.

For clarity, the findings are presented in two parts: first, the key factors identified in the SWOT analysis, followed by the strategic recommendations derived via the TOWS matrix.

#### Strengths

The included studies highlighted multiple internal facilitators of effective clinical audits. A primary strength is improved clinical care processes and outcomes audits promote adherence to evidence-based guidelines, an alignment linked to safer practices and better patient outcomes.^13 14^ Many studies also emphasised knowledge sharing and teamwork fostered by audits: in supportive, blame-free environments, regular audit meetings allow interdisciplinary learning and open dialogue, enhancing professional development and staff morale.^8^ This collaborative approach enhances communication across departments by breaking down hierarchies and silos, thereby building trust and a shared culture of continuous improvement.^15^ Importantly, audits often act as a catalyst for change by identifying gaps and prompting interventions (like new training or protocols), which subsequently improve care processes.^4^ Over time, such audit-driven improvements have translated into higher patient satisfaction, for instance by reducing waiting times or standardising follow-up communications.^6^

#### Weaknesses

Several internal barriers were identified that can undermine audit effectiveness. Diminished clinical ownership and engagement was commonly reported; when clinicians perceive audits as bureaucratic or punitive, motivation to implement changes declines, leading to poor uptake of audit recommendations and limited impact on practice change.^9^ A related weakness is a prevailing culture of fear or blame; in some settings, clinicians are hesitant to report errors or discuss deficiencies openly due to concerns about legal repercussions, professional criticism or punitive consequences.^16^

Hierarchical and territorial barriers also hinder audit processes. Rigid power dynamics and departmental silos can suppress open communication, discourage junior staff from challenging established practices and result in teams prioritising local performance metrics over organisation-wide learning and improvement.^11^

Resource constraints were another major weakness. Many audits operate with limited staffing, funding or protected time, forcing audit activities to compete with routine clinical duties. As a result, audits often focus narrowly on immediate high-risk issues, while broader or chronic care quality problems remain unaddressed.^10^ For example, when resources are restricted, audits may concentrate on urgent patient-safety domains (such as infection control), leaving longer-term areas like chronic disease management without systematic follow-up.^10^

Inadequate information systems further compounded these challenges. Fragmented, paper-based or outdated data systems made data collection and analysis labour-intensive, error-prone and slow, reducing both clinician confidence in audit findings and the timeliness of feedback needed to drive improvement.^9^

Critically, a recurring weakness across the included literature was failure to complete the full audit cycle. While many studies reported baseline measurement and initial feedback, relatively few documented a subsequent re-audit or repeat measurement to verify whether implemented changes led to sustained improvement. This failure to ‘close the audit loop’ represents a fundamental breakdown in audit methodology. Without scheduled re-measurement, improvements remain unverified, accountability is weakened and gains are vulnerable to regression over time.^6 12^ The omission of re-audit thus emerged as a significant internal limitation that undermines the long-term effectiveness of clinical audit programmes.

#### Opportunities

Multiple external factors emerged as opportunities to strengthen clinical audit programmes. One key opportunity is leveraging clinical information systems: integrating audit findings into electronic health records (for example, adding mandatory documentation fields or automated reminders based on audit insights) can improve data quality and directly embed quality checks into care processes.^1^ Another opportunity is policy development informed by audit evidence using audit results to guide new or updated organisational policies and protocols (eg, addressing recurrent delays or safety issues) helps ensure improvements are institutionalised.^4^ Targeted skill development is also an opportunity: the literature noted that audits often reveal staff knowledge gaps, which organisations can address through focused training programmes or professional development initiatives aligned with the identified needs.^8^ Additionally, audits highlight resource use inefficiencies, creating opportunities for resource optimisation (such as reducing redundant tests or reallocating staff) to improve cost-effectiveness and care delivery.^17^ Audits even enable development of local standards tailored to an institution’s context; by pinpointing where generic guidelines do not align with local needs, audit findings can inform more relevant, site-specific standards that boost clinician buy-in and compliance.^18^ Finally, strengthening accountability is an opportunity: transparent audit processes and feedback loops promote a sense of shared responsibility for quality, encouraging clinicians to uphold standards consistently.^19^

#### Threats

The analysis identified several external challenges that threaten audit success. Scepticism and resistance to change among staff or leadership can undermine audits—clinicians may doubt the value of audits or view them as burdensome if past efforts did not show clear benefits, which can diminish engagement.^10^ A closely related threat is a prevailing culture of blame or intimidation: without psychological safety, clinicians fear humiliation or punishment, leading to superficial participation or avoidance of audits altogether.^12 20^ Institutional inertia poses another threat even when audits identify needed improvements, bureaucratic delays or lukewarm leadership support can stifle the implementation of changes, turning audits into a perfunctory exercise rather than a catalyst for improvement.^20^ Time constraints in busy healthcare settings also jeopardise audit initiatives; without protected time, clinicians juggling heavy workloads may find it difficult to consistently conduct or follow through on audits.^7^ This challenge, if unaddressed, can cause audits to be rushed or deferred, eroding their effectiveness.^20^ Additionally, professional isolation and burnout can result if a few individuals shoulder the audit workload without adequate support, reducing their motivation and continuity in quality improvement efforts.^8^

### TOWS-derived strategic recommendations

Building on the SWOT findings, a TOWS matrix (figure 3) was constructed to propose actionable strategies that leverage strengths and opportunities or mitigate weaknesses and threats. The analysis generated four sets of strategies—S–O, W–O, S–T and W–T—each directly informed by specific SWOT factors identified above.

- S–O (Strength–Opportunity) strategies: leverage internal strengths to exploit external opportunities. For example, healthcare teams can use their strong interdisciplinary collaboration and prior audit successes (strengths) to integrate audit insights into electronic systems and policymaking (opportunities).^15 20^ By capitalising on robust teamwork and showcasing evidence of improved patient outcomes, this strategy embeds audit-driven improvements into routine practice and wider organisational initiatives.
- W–O (Weakness–Opportunity) strategies: use external opportunities to address internal weaknesses. For instance, introducing targeted training and user-friendly audit software (opportunities) can improve clinician engagement and data accuracy (addressing weaknesses like skill gaps and inefficient processes).^8 10^ Similarly, obtaining dedicated funding or sharing resources across departments (opportunities) can help overcome staffing and resource shortages (weaknesses) that currently limit the scope and frequency of audits.^10^
- S–T (Strength–Threat) strategies: deploy strengths to counteract external threats. One strategy is to publicise audit-driven improvements and nurture clinician ‘audit champions’ (using strengths such as proven care improvements and enthusiastic staff) to reduce scepticism and inertia at the organisational level (threats).^13 20^ By highlighting success stories and involving respected team members, healthcare organisations can build trust in the audit process, counter resistance to change and secure stronger leadership support.
- W–T (Weakness–Threat) strategies: minimise internal weaknesses to lessen external threats. For example, fostering a non-punitive, transparent audit culture (addressing weaknesses like fear of blame and poor communication) can mitigate the threat of staff disengagement or intimidation.^16 20^ This involves clearly communicating confidentiality protections and improvement-focused goals, so clinicians feel safe to participate fully. Likewise, providing protected time and team support for audit activities (tackling weaknesses around time pressure and isolation) helps maintain engagement and momentum even in the face of heavy clinical workloads (threat).^7 8^

Each of these TOWS strategies is directly grounded in the evidence collected from our systematic review. By linking specific internal and external factors, the TOWS matrix offers a structured roadmap that researchers and practitioners can emulate, aiming to enhance clinical audits as effective drivers of continuous quality improvement.

### Strategic recommendations

Building on the TOWS analysis, we propose the following key recommendations for healthcare organisations to maximise the impact of clinical audits: integrate audit outcomes into clinical information systems (with features like mandatory fields, alerts and decision support) to promote real-time adherence to best practices, a step shown to reduce errors and enhance efficiency.^21^ Foster a culture of psychological safety by implementing non-punitive audit policies, assuring confidentiality and even allowing anonymous input, so clinicians can participate without fear, encouraging honest, transparent engagement.^22 23^ Invest in workforce development through targeted micro-learning and simulations based on audit findings; ensure clinicians have protected time for these educational activities and consider appointing dedicated audit coordinators to manage workloads and sustain participation.^8^ Align governance and policy with audit results by routinely updating protocols (for infection control, medication management, discharge planning, etc) in response to audit evidence, to ensure improvements are institutionalised.^4^ Use data dashboards and audit ‘champions’ to maintain momentum visualising progress and having respected staff advocate for audit-driven change can address scepticism and help overcome organisational resistance.^10 24^ Anticipated outcomes of these strategies include fewer medication errors, better compliance with care standards, higher patient satisfaction, more efficient use of resources, and a stronger culture of interdisciplinary teamwork and shared accountability.

## Discussion

This analysis confirms that clinical audits are instrumental in improving guideline compliance, fostering interdisciplinary teamwork and enhancing patient satisfaction, in line with earlier.^5 6^ We identified significant strengths such as improved clinical care processes, increased knowledge sharing and stronger teamwork dynamics resulting from audit activities. At the same time, we found persistent weaknesses including reduced clinical ownership of audit processes, fear of litigation among practitioners and shortcomings in information systems. The TOWS analysis underscored strategies like adopting non-punitive frameworks, engaging leadership and maintaining open communication as critical for overcoming threats like widespread scepticism, institutional inertia and limited time.

Our findings are consistent with previous studies demonstrating the positive impact of clinical audits on healthcare quality and outcomes. For example, prior work has shown improvements in care quality and adherence to guidelines through audit initiatives^5^ and improved organisational behaviour via audit feedback.^6^ Similarly, well-documented barriers such as resource constraints and hierarchical challenges^11^ emerged in our review. By employing the SWOT and TOWS frameworks, our study adds to the literature by providing a structured approach to translate these observations into a coherent set of actionable recommendations for optimising clinical audit processes.

Overall, this study offers a comprehensive evaluation of the role and impact of clinical audits in healthcare management. Using SWOT and TOWS, we systematically identified internal and external factors that influence the effectiveness of audits. Our findings reinforce that clinical audit is a cornerstone of quality improvement, patient safety and organisational learning in modern healthcare systems.

### Generalisability and adaptation to low-resource settings

While our review encompassed diverse healthcare settings, the high-income dominance of the evidence base means that the SWOT and TOWS-derived strategies should be applied judiciously outside such contexts. For instance, one of our key recommendations is to integrate audit outcomes into electronic health record (EHR) systems to institutionalise improvements. Yet in many LMIC healthcare facilities, comprehensive EHR systems are lacking or remain in early stages of implementation; in these cases, an alternative preliminary strategy might involve strengthening basic health information infrastructure or employing simplified, paper-based audit tools as a foundation. Similarly, the strategy of assigning dedicated audit coordinators or allocating protected staff time for audits, while feasible in well-resourced institutions, may be unrealistic in severely understaffed clinics; resource-constrained settings might need to prioritise task-shifting, external support or integration of audit activities into routine workflows until additional workforce capacity and funding are available. By explicitly noting these distinctions, we ensure that our recommendations are not overgeneralised to settings with substantially different resource profiles. We emphasise that the overall principles of our SWOT–TOWS strategies such as fostering a non-punitive culture and using audit data for feedback and policy remain widely relevant, but implementers in LMIC contexts should adapt these strategies to local conditions. We now discuss these nuances in the manuscript to guide readers from diverse settings in interpreting and applying our findings appropriately.

### Integrating strengths and opportunities

The evidence shows that clinical audits can drive significant improvements in care by aligning practice with evidence-based standards, reducing errors (especially medication-related errors) and cultivating continuous learning cultures.^13 14^ Regular audit meetings and the ensuing interdisciplinary collaboration not only improve professional satisfaction but also break down hierarchies, promoting open dialogue and shared accountability among healthcare providers.^2 8 15^ Embedding audit output into CIS (an opportunity) allows real-time compliance monitoring and rapid feedback, which further reinforces adherence to best practices.^1^ Developing local standards from audit insights ensures that quality improvement measures are tailored to the organisation’s specific context, making them more effective and sustainable.^18^ Together, leveraging these strengths and opportunities can magnify the benefits of audits.

### Addressing weaknesses and threats

Despite the advantages, several challenges threaten the effectiveness of clinical audits. We observed issues like diminished clinical ownership of audits, fear of litigation or blame, rigid organisational structures and resource limitations factors well-documented in the literature.^9–11 16^ These can lead to disengagement, under-reporting of issues and resistance to adopting audit recommendations. The TOWS strategies we outlined suggest ways to mitigate such weaknesses: implementing non-punitive frameworks and robust confidentiality policies to address fear of reporting, providing protected time and dedicated personnel to ensure audits are carried out thoroughly and upgrading information systems to support data needs.^16 25–27^ To confront external threats like scepticism, reluctance to critique peers and institutional inertia, it is essential to cultivate psychological safety and clear communication.^12 20 28^ Strong leadership commitment, along with empowering audit champions, is crucial to drive cultural change and embed audit-driven improvements into routine practice.^24 29^

### Implications for practice

Our recommendations translate these findings into practical steps for healthcare managers and policymakers. Integrating audit results into Clinical Information System (CIS) and standardising feedback loops can be implemented to immediately enhance compliance and facilitate learning.^1 4 29^ Tying audit outcomes to policy development means that the insights gathered do not remain on paper but lead to tangible changes in procedures and guidelines, improving patient safety and resource use.^6 17^ Involving clinicians in every stage of the audit process from design to implementation to interpretation ensures that the resulting recommendations are realistic, relevant and more readily accepted.^20^ Linking audit outcomes with targeted training addresses any identified knowledge gaps and visibly invests in professional growth, which reinforces a continuous improvement ethos.^8^ In essence, healthcare institutions should treat audit programmes not as isolated quality checks but as integral to their operations and development strategies.

### Implementation science grounding of strategies

Our TOWS-derived recommendations have been explicitly anchored to established implementation science frameworks to enhance their theoretical validity. For instance, our strategy emphasising psychological safety and a blame-free culture within audit teams aligns with the Inner Setting constructs of the CFIR notably, implementation climate and learning climate (a dimension of culture that supports open dialogue and experimentation).^30^ Similarly, the recommendation to employ clinical audit ‘champions’ and data dashboards to reinforce continuous feedback loops corresponds to well-recognised strategies in the ERIC taxonomy specifically, identifying and preparing champions and audit-and-feedback interventions.^31^ Furthermore, our suggestion to align organisational governance and policy with audit insights reflects critical Outer Setting factors (eg, external policies and incentives) described by CFIR^30^ and resonates with Grol and Grimshaw’s implementation model that emphasises involving leadership and aligning system incentives to sustain practice change.^32^ By drawing these parallels, we underscore that our strategic guidance is consistent with and reinforced by core implementation science concepts, thereby bridging our evidence-based SWOT–TOWS analysis with widely accepted theory on how to effect change in healthcare settings.

### Contextualising strategies by resource readiness

It is also important to differentiate our recommendations based on implementation readiness and resource context. Some strategies identified (eg, integrating clinical audits with advanced EHR systems or automating data collection processes) presume a level of infrastructure and capacity typically found in well-resourced settings. We now distinguish such ‘advanced’ strategies as longer-term or aspirational steps for environments where robust health IT and funding exist (common in high-income health systems). In contrast, other ‘foundational’ strategies (eg, establishing basic audit feedback meetings, nurturing a non-punitive culture, leveraging simple data forms) are immediately actionable in most settings and do not require significant new resources. For example, while linking audit processes to an integrated EHR can greatly streamline data monitoring in a technologically mature primary care system, many low-income and middle-income clinics still lack electronic records and rely on paper-based systems.^33^ In those contexts, a more feasible near-term approach would be to use paper or mobile-based data tools and manual feedback charts as precursors to full EHR integration. Likewise, a recommendation to ‘align governance structures with audit results’ might entail formal policy mandates or dedicated quality units feasible in health systems with mature governance whereas in resource-constrained settings, this strategy may first require capacity building and external support (eg, leadership training or non-governmental organization (NGO) partnerships). We have revised our strategic recommendations to explicitly indicate which actions are resource-intensive versus those that can be implemented under existing constraints, helping stakeholders in various settings identify context-appropriate steps. This stratified approach ensures that our TOWS matrix output is practical and adaptable across diverse primary care environments.

### Positioning novelty and value of SWOT–TOWS lens

Our analysis builds on and differs from prior syntheses in the audit-and-feedback domain. Previous systematic reviews, including a comprehensive Cochrane review by Ivers and colleagues,^29^ have examined audit and feedback interventions and identified factors influencing their effectiveness. Those reviews applied traditional narrative or quantitative approaches (eg, meta-analysis and meta-regression) to aggregate results and pinpoint what elements of audit and feedback are associated with improved clinical practice. By contrast, the present review introduces a strategic planning lens (SWOT–TOWS) to this evidence. Specifically, we categorise reported enablers and barriers of clinical audit into internal ‘Strengths’ and ‘Weaknesses’ versus external ‘Opportunities’ and ‘Threats.’ This structured mapping helps differentiate between factors that are intrinsic to the implementation setting (eg, leadership support or data system quality) and those arising from the broader environment (eg, policy mandates or resource constraints). Moreover, through the TOWS matrix, we systematically pair these factors to devise actionable strategies—for example, using identified strengths (such as strong clinician engagement) to exploit opportunities (like external training programmes), or addressing weaknesses (eg, limited Information Technology (IT)infrastructure) by mitigating threats (eg, lack of funding) with targeted solutions. This approach provides a novel contribution by moving beyond descriptive synthesis: it offers a strategic integration of the literature, effectively bridging the gap between evidence and practice by generating contextually tailored implementation recommendations. We have tempered our claims of novelty to reflect this nuance and to acknowledge that many of the themes we identified align with findings from earlier audit-and-feedback reviews (eg, the importance of leadership support, feedback timeliness, IT systems, etc), as noted by Ivers *et al*.^29^ The difference lies in how we organise and translate these findings. Our SWOT–TOWS lens yields a holistic viewpoint akin to a strategic roadmap for enhancing clinical audit processes which traditional narrative or meta-analytic reviews, by design, do not provide. We believe this structured, translational perspective complements the existing audit-and-feedback literature and can help stakeholders better apply the collective insights from prior studies in a practical, context-aware manner.

### Limitations and future directions

This study is based on a structured synthesis of existing literature, which may limit the generalisability of the findings. We depended on the scope and quality of available studies; thus, some contextual nuances might not be fully captured. Future research could employ longitudinal designs or mixed-method approaches (combining quantitative outcome tracking with qualitative insights) to evaluate the long-term impact of implementing the recommended strategies. Additionally, exploring the perspectives of patients and frontline staff through interviews or surveys could provide a more nuanced understanding of how audits influence care and where gaps remain. The evolving landscape of digital health and advanced analytics also presents new opportunities to enhance clinical audits future studies should examine how tools like real-time data dashboards, machine learning for pattern detection and mobile audit platforms might improve efficiency and effectiveness.^10 20^ Finally, our search strategy did not include grey literature (eg, unpublished reports, theses or conference proceedings). As a result, relevant data or implementation insights outside the peer‐reviewed literature may have been missed, introducing a risk of publication bias in our evidence base.

### Limited representation of LMIC healthcare and generalisability

Our evidence base is geographically and economically skewed toward well-resourced health systems, which may limit the transferability of findings to lower-resourced settings. As noted above, only ~6% of the included studies were from lower–middle–income countries and none were from low-income countries, whereas almost three-quarters were conducted in high-income settings. This imbalance likely reflects the historical trend that much of the clinical audit literature originates from high-income healthcare systems (eg, the UK National Health Service (NHS), Australia or Gulf Cooperation Council countries) where structured audit programmes, robust health information systems and formal clinical governance frameworks are well-established. Primary care contexts in low-income or lower–middle–income countries often operate under qualitatively different constraints that go beyond simply being ‘more intense’ versions of HIC challenges. For example, many LMIC primary care settings rely on paper-based or fragmented health record systems instead of integrated electronic health records, face severe healthcare workforce shortages, high staff turnover (with reliance on rotating or volunteer staff), limited protected time for quality improvement, and often have weaker or nascent clinical governance structures and support. These contextual differences can fundamentally alter the feasibility and impact of clinical audits. We therefore explicitly caution that our SWOT analysis and ensuing TOWS strategic recommendations, derived largely from higher-resource environments, may not fully capture or directly apply to the realities of resource-constrained primary care settings without adaptation. In recognition of this limitation, we have added text in the Discussion to highlight where certain strategies might need tailoring or additional steps before they can be effective in LMIC contexts (see below). Future research should specifically target LMIC primary care contexts to validate and extend our findings under markedly different system conditions.

### Limitations (contextual feasibility and adaptation)

Finally, we emphasise that context matters greatly in the application of our findings. The evidence base of our review was predominantly drawn from higher-income healthcare systems, which inherently offered stronger infrastructural and institutional support for clinical audits. As such, some of our synthesised Strengths, Weaknesses, Opportunities, and Threats and the strategies derived from them may not directly translate to lower-resource settings without adaptation. We caution readers, for example, that recommendations like implementing sophisticated electronic data systems or formal governance feedback loops presume conditions (stable funding, technical infrastructure, management structures) are often absent in rural or resource-limited primary care clinics. Adaptation is crucial: strategies should be tailored to an institution’s readiness and context. By explicitly noting these limitations and context-specific considerations, we encourage implementers, especially in LMIC settings, to treat our TOWS strategies as a menu of options to be selected and modified according to local feasibility, rather than as one-size-fits-all solutions. Future implementation research should also prioritise testing these strategies in varied settings to confirm their effectiveness and to refine them for different health system contexts.

## Conclusion

Clinical audit remains a potent instrument for driving quality improvement in healthcare. By leveraging its strengths and available opportunities while proactively addressing weaknesses and guarding against threats organisations can transform audits from mere compliance exercises into dynamic tools for organisational learning and patient-centred care. The strategic recommendations derived from our SWOT and TOWS analyses offer a clear roadmap for optimising audit processes, strengthening clinician engagement and achieving sustainable improvements in clinical practice. Ultimately, when integrated thoughtfully, clinical audits can continuously inform and elevate the standard of care, fostering an environment of accountability, collaboration and excellence in healthcare delivery.

## Supplementary material

10.1136/bmjoq-2026-004162 online supplemental table 1

10.1136/bmjoq-2026-004162 online supplemental table 2

10.1136/bmjoq-2026-004162 online supplemental table 3

## Data Availability

Data are available upon reasonable request.
