# Supplementary material for: Clinical audit as a catalyst for quality improvement in healthcare systems: strategic insights from a systematic review and SWOT–TOWS analysis
Source: BMJ Open Qual. 2026 Oct 21;15(4):e004162. doi: 10.1136/bmjoq-2026-004162 (PMC13691825; doi:10.1136/bmjoq-2026-004162)
Supplement: Supplementary file 1 [file bmjoq-15-4-s001.pdf]

Supplementary Table 1: Full Search Strategies (2015–2024)

Below are the complete search strategies used in each database, including all Boolean operators, synonyms, and limits (date range and language). Each strategy was executed to retrieve literature on clinical audits and quality improvement, incorporating terms related to internal/external factor analysis (SWOT/TOWS). All searches were limited to English language and publication years 2015 through March 2024.

- **PubMed (MEDLINE) search strategy:**

1	("Medical Audit"[MeSH] OR "clinical audit"[All Fields] OR "clinical audits"[All Fields] OR "audit cycle"[All Fields])
2	AND ("Quality Improvement"[MeSH] OR "Quality of Health Care"[MeSH] OR "quality improvement"[All Fields] OR "quality assurance"[All Fields] OR "patient safety"[All Fields] OR "evidence-based practice"[All Fields] OR "continuous monitoring"[All Fields] OR "benchmarking"[All Fields] OR "performance indicators"[All Fields] OR "stakeholder engagement"[All Fields] OR "change management"[All Fields])
3	AND ("SWOT"[All Fields] OR "SWOT analysis"[All Fields] OR "strengths weaknesses opportunities threats"[All Fields] OR "TOWS"[All Fields] OR "TOWS matrix"[All Fields])
4	Filters: Publication date from 2015/01/01 to 2024/03/31; English; Humans.

- **Web of Science search strategy:**

1	TS = (("clinical audit" OR "audit cycle" OR "medical audit")
2	AND ("quality improvement" OR "quality of care" OR "patient safety" OR "evidence-based practice" OR "benchmarking" OR "continuous monitoring" OR "performance indicators" OR "stakeholder engagement" OR "change management")
3	AND (SWOT OR "SWOT analysis" OR TOWS OR "TOWS matrix"))
4	Refined by: Publication Years = 2015–2024; Languages = English.

- **Cochrane Library search strategy:**

1	("clinical audit" OR "medical audit" OR "audit programme")
2	AND ("quality improvement" OR "quality assurance" OR "patient safety" OR "benchmarking" OR "performance indicator" OR "evidence-based practice")
3	AND (SWOT OR TOWS OR "strengths and weaknesses")

Classification:
PUBLIC

4	Limits applied: 2015–2024, English, publication types (all available).

- **Mendeley (literature search) strategy:**

1	("clinical audit" AND "quality improvement") AND (SWOT OR TOWS OR "stakeholder engagement" OR "change management" OR "patient safety" OR "evidence-based practice")
2	Filter: Year 2015–2024; English.

Note: The above queries reflect the intended search logic grouping five conceptual domains (clinical audit; healthcare quality improvement; SWOT analysis; TOWS matrix; process/outcome factors). Minor syntax adjustments were made per database (e.g., MeSH/field tags in PubMed, TS= in Web of Science). These comprehensive strategies were developed to ensure capture of relevant literature aligning clinical audits with quality improvement and strategic factor analysis, following PRISMA guidelines.

Classification:
PUBLIC
