# Supplementary material for: Clinical audit as a catalyst for quality improvement in healthcare systems: strategic insights from a systematic review and SWOT–TOWS analysis
Source: BMJ Open Qual. 2026 Oct 21;15(4):e004162. doi: 10.1136/bmjoq-2026-004162 (PMC13691825; doi:10.1136/bmjoq-2026-004162)
Supplement: Supplementary file 2 [file bmjoq-15-4-s002.pdf]

Supplementary table 2: Relevance of included study types for SWOT analysis.

Type of Included Studies	Primary Focus Area	Relevance to SWOT/TOWS Analysis
**Quantitative Outcome Studies (e.g., audits with pre/post data)**	Measured objective outcomes of clinical audit interventions using quantitative data (e.g., improvements in guideline adherence, reduction in complication rates, changes in patient or process outcomes). Often included statistical analyses of performance indicators before vs. after audits.	Provided evidence of the benefits and effectiveness of clinical audits, supplying support for Strengths and potential Opportunities. For example, multiple studies showed that audits led to significantly improved compliance with care standards and optimized resource use. Such data underpin identified Strengths (e.g., "audits improve quality of care") and justify strategic Opportunities (e.g., expanding audit programs to leverage proven gains).
**Qualitative Audit Process Studies (e.g., interviews, surveys)**	Explored the experience and process of conducting audits, often through interviews, focus groups, or surveys with healthcare professionals. Examined factors like staff perceptions, cultural and organizational dynamics, communication during audits, and common barriers/facilitators in implementation (e.g., time constraints, fear of blame, teamwork culture).	Offered insight into practical challenges and informed Weaknesses and Threats. These studies uncovered frequent obstacles (insufficient resources, resistance to feedback, poor data systems, etc.). Such findings translated directly into the SWOT analysis as internal weaknesses or external threats that hinder audit effectiveness. They also guided TOWS strategies by highlighting issues to be mitigated (e.g., recommending non-punitive audit processes to address fear of criticism).
**Mixed-Methods & Case Studies (combined quantitative–qualitative)**	Provided in-depth case examples of clinical audit initiatives using a mix of outcome data and contextual analysis. For instance, a case study might report quantitative	Illustrated how audits work in practice, bridging outcomes with context. Such studies reinforced Strengths by showing real world improvements (validating that audits can be effective) while also revealing Weaknesses/Threats encountered (e.g., initial staff skepticism, workflow hurdles)

Classification: PUBLIC

	improvements from an audit (e.g., reduced infection rates) alongside qualitative insights into how the audit was implemented and what contextual factors influenced success or failure. Often focused on single institutions or specific programs, offering a holistic view of an audit's impact.	and how they were overcome. These examples were valuable for the TOWS analysis: they provided practical lessons on leveraging strengths to seize opportunities (e.g., how a successful audit program capitalized on strong teamwork) and on addressing weaknesses to mitigate threats (e.g., how leadership support helped overcome resistance).
**Healthcare Management–Focused Studies (systems/policy level)**	Examined audits from an organizational or system-wide perspective. Instead of focusing on a single audit's outcome, these studies looked at how audit programs integrate into broader healthcare management, such as alignment with quality improvement policies, leadership engagement, or health system performance. Some analyzed institutional audit frameworks or how audit findings inform management decisions and policy updates.	Informed the external perspective of SWOT (especially Opportunities and Threats) by highlighting how audits interact with the wider system. For example, some studies discussed using audit results to drive policy changes or improve clinical governance, contributing to SWOT Opportunities (e.g., "use audit data for policy development"). Others noted high-level obstacles such as lack of organizational support, reinforcing identified Threats (e.g., institutional inertia). These insights ensured that our TOWS strategies considered not just clinical team-level actions but also system-wide measures for instance, recommending integration of audit findings into hospital policy and information systems was based on evidence from these studies.
**Studies Highlighting SWOT Factors (reviews of audit barriers/enablers)**	Explicitly identified strengths, weaknesses, opportunities, or threats related to clinical audits by synthesizing previous findings. This category includes literature reviews or analyses cataloguing	Directly fed into our SWOT analysis with validated factors. For instance, systematic reviews of organizational barriers (like hierarchical issues or resource gaps) helped confirm several SWOT Weaknesses/Threats. Similarly, papers emphasizing known advantages of audits or conditions for success reinforced SWOT Strengths and

	common barriers to successful audits, critical success factors, or lessons learned across many settings. They provided ready lists of what tends to go right or wrong in audit initiatives.	suggested Opportunities. Because these studies explicitly dealt with audit pros and cons, they ensured our SWOT/TOWS was comprehensive and evidence based. The TOWS strategies often drew on these findings e.g., if a review highlighted “lack of management support” as a threat, our strategy matrix addressed it by proposing stronger leadership engagement in audits.
