# Supplementary material for: Clinical audit as a catalyst for quality improvement in healthcare systems: strategic insights from a systematic review and SWOT–TOWS analysis
Source: BMJ Open Qual. 2026 Oct 21;15(4):e004162. doi: 10.1136/bmjoq-2026-004162 (PMC13691825; doi:10.1136/bmjoq-2026-004162)
Supplement: Supplementary file 3 [file bmjoq-15-4-s003.pdf]

Supplementary Table 3: Provides illustrative examples of how extracted themes were mapped to SWOT categories.

Theme / Finding	SWOT Category	Rationale
"Routine clinical audits significantly improved adherence to national care guidelines." (Multiple studies)	Strength (Internal Positive)	Demonstrates an internal success of audit processes (better care quality), an inherent advantage of audit practice.
"Staff have limited time to conduct audits due to workload." (Several studies)	Weaknesses (Internal Negative)	Reflects a resource limitation within the organization (insufficient protected time), an internal barrier undermining audit execution.
"Integration of audit data into electronic health records enables continuous monitoring." (Single study)	Opportunity (External Positive)	An external enhancement that can be leveraged (advanced health IT infrastructure), presenting a chance to augment audit impact.
"Lack of senior management support for audit results in poor uptake of changes." (Several studies)	Threat (External Negative)	Indicates a broader organizational context issue (leadership climate) that hinders audit success, beyond the direct control of the audit team.

Following categorization, the review team compiled the final list of SWOT factors with supporting references. This evidence base informed the subsequent TOWS matrix, in which Strengths, Weaknesses, Opportunities and Threats were paired to generate practical, evidence-informed strategies for strengthening clinical audit implementation. Reviewer triangulation, consensus discussion and documentation of decisions helped ensure that the analytical process was systematic, coherent and verifiable.

Classification:
PUBLIC
